# Fertility preservation in women with diminished ovarian reserve: evaluating the AMH criteria

**DOI:** 10.1007/s10815-025-03779-w

**Published:** 2025-12-26

**Authors:** Maria Elisabetta Coccia, Benedetta Gabbrielli, Giulia Cutajar, Francesca Piazzini, Paolo Evangelisti, Laura Badolato, Patrizia Falcone, Rossella Fucci, Carlo Bulletti

**Affiliations:** 1 Department of Biomedical, Experimental, and Clinical Sciences Mario Serio, University of Florence, Largo Brambilla, 3, 50134 Florence, Italy; 2 Assisted Reproductive Technology Centre, Careggi University Hospital, 50134 Florence, Italy; 3 Department of Obstetrics, Gynecology and Reproductive Sciences, Yale University, New Haven, CT USA

**Keywords:** Oocyte cryopreservation, Vitrification, Diminished ovarian reserve, DuoStim, Reproductive planning, Ovarian stimulation

## Abstract

**Purpose:**

Does fertility preservation (FP) through oocyte cryopreservation provide realistic reproductive opportunities for diminished ovarian reserve (DOR)? Literature suggests cumulative live birth rates of 30–45% with 8–10 oocytes under 35 years old. Insufficient data exist to define whether DOR patients should be offered FP systematically.

**Methods:**

This retrospective single-center study analyzed data from 304 women undergoing oocyte cryopreservation (January 2016–December 2024). Patients were stratified into cohort 1 (Anti-Müllerian hormone—AMH—≤ 0.5 ng/mL, *n* = 49) and cohort 2 (AMH > 0.5 ng/mL, *n* = 255). Primary outcomes included retrieved oocytes (RO) and vitrified oocytes (VO). Secondary outcome examined DuoStim results. Statistical analysis included correlation assessments, ANCOVA, and multiple linear regression.

**Results:**

DOR patients achieved lower oocyte yields compared to cohort 2 (RO: 3.1 ± 2.3 vs. 9.0 ± 6.5; VO: 2.3 ± 1.9 vs. 7.4 ± 5.1; *p* < 0.001), despite higher gonadotropin doses. AMH strongly correlated with RO (*ρ* = 0.636, *p* < 0.001) and VO (*ρ* = 0.624, *p* < 0.001). Linear regression confirmed AMH (*B* = 1.151, *p* < 0.001) and age (*B* =  − 0.139, *p* = 0.002) as significant predictors of VO. In DuoStim subgroup, DOR patients achieved 3.3 ± 2.1 total VO compared to 6.9 ± 3.3 in cohort 2 (*p* = 0.001).

**Conclusion:**

DOR patients achieve oocyte yields substantially below thresholds associated with reasonable live birth rates, raising concerns regarding FP efficacy. These findings highlight the need for personalized counseling that considers individual patient characteristics and provides evidence-based, realistic expectations for FP. A revision of current AMH thresholds may improve patient selection and cost-effectiveness of FP programs. Younger DOR patients may benefit from oocyte cryopreservation for FP, emphasizing the importance of age stratification.

## Introduction

Fertility preservation (FP) includes treatments and techniques intended to protect future reproductive potential reducing infertility risk associated with various conditions and medical treatments. The development of female FP was primarily driven by the following: (1) advances in cancer treatments and survival rates that highlighted the importance of long-term quality of life among survivors [7, 12] and (2) advances in oocyte cryopreservation techniques.

While the first live birth from slow freezing protocols was reported in 1986 [4], the real breakthrough came with vitrification, a rapid freezing method which markedly improved oocyte survival and developmental potential, leading to the first live birth in 1999 [15]. In 2012, the American Society for Reproductive Medicine removed the experimental designation from mature oocyte cryopreservation. FP is now a critical aspect of cancer care, and has expanded to additional medical indications and to elective FP in women seeking to delay childbearing [6].

Diminished ovarian reserve (DOR) poses specific challenges for FP and assisted reproductive technologies (ART) in general [24], with considerable healthcare and economic implications. DOR may reflect the natural, age-related decline in both quantity and quality of ovarian follicles or result from non-age-related factors, including idiopathic causes [1, 10, 14]. DOR is frequently associated with poor ovarian response (POR) to controlled ovarian stimulation (COS) [3, 27]. Serum Anti-Müllerian Hormone (AMH) concentration strongly correlates with ovarian follicles number, and is considered a reliable biomarker of ovarian reserve [17].

Standard cryopreservation protocols involve COS during the follicular phase, followed by oocyte retrieval once maturation is achieved. The DuoStim protocol, first reported in 2013, involves two consecutive stimulations within the same menstrual cycle, during follicular and luteal phases, to improve oocyte yield [26].

This study compares conventional stimulation outcomes in women with AMH ≤ 0.5 ng/mL versus AMH > 0.5 ng/mL. In addition, an observational descriptive analysis was performed on patients undergoing DuoStim following a suboptimal follicular phase response, in order to provide real-world insights into its potential value in this challenging population.

The aim is to evaluate whether oocyte cryopreservation remains a clinically meaningful option for DOR patients, providing useful insights for personalized counseling.

## Methods

We reviewed data from a continuously updated, prospectively maintained institutional database, of patients presenting to the Fertility Preservation Outpatient Clinic of Careggi University Hospital, a single tertiary-level ART Center, between January 7, 2016, and December 31, 2024. All patients presenting at the initial consultation were considered for enrollment. Eligible participants were women aged ≤ 40 years, in accordance with local healthcare policy for publicly funded FP. Exclusion criteria included lack of informed consent, discontinuation of FP process prior to oocyte retrieval, or absence of recent AMH measurements.

**Fig. 1:**
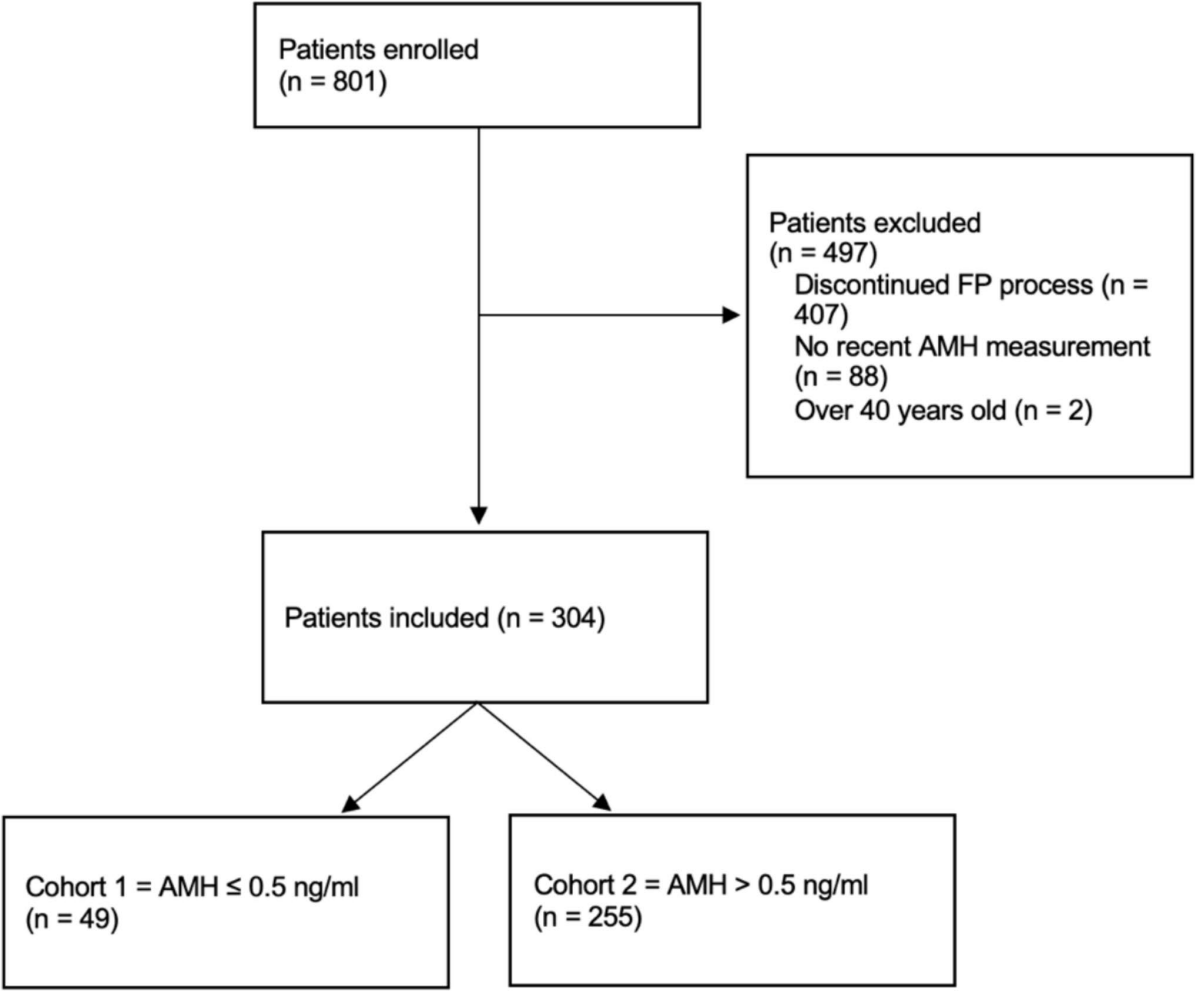
Patient enrollment and cohort allocation flowchart. The study enrolled 801 patients, of whom 497 were excluded due to discontinued FP process (*n* = 407), absence of recent AMH measurement (*n* = 88), or age over 40 years (*n* = 2). The final study population consisted of 304 patients, divided into two cohorts based on AMH levels: cohort 1 with AMH ≤ 0.5 ng/mL (*n* = 49) and cohort 2 with AMH > 0.5 ng/mL (*n* = 255). AMH, anti-Müllerian hormone; FP, fertility preservation

The final analysis included 304 patients (mean age of 31.6 ± 5.5 years; range 16–40), divided into two cohorts based on AMH levels: AMH ≤ 0.5 ng/mL (cohort 1, DOR patients) and AMH > 0.5 ng/mL (cohort 2, control group). The 0.5 ng/mL threshold, identified by the Bologna criteria (2011) as a high-specificity cut-off for DOR, has been used in our setting since 2015 to determine eligibility for publicly funded FP [9]. A flow diagram of patient inclusion is provided in Fig. 1.

The study was approved by the local Ethics Committee (15,404 CAM-BIO) and conducted according to Declaration of Helsinki STROBE guidelines.

All patients underwent COS using standard gonadotropin protocols, including recombinant FSH (r-FSH), recombinant LH (r-LH), human menopausal gonadotropin (hMG), or combinations. Pituitary suppression was achieved using gonadotropin releasing hormone (GnRH) antagonists or agonists. In breast cancer patients, letrozole was co-administered to minimize estrogen exposure. Mean stimulation duration was 10.0 ± 2.0 days (range: 4–18).

A luteal phase stimulation (LPS) following the follicular phase stimulation (FPS) within the same menstrual cycle (DuoStim) was employed in a subgroup of patients (*n* = 38) who failed to achieve adequate oocyte yield after FPS.

Ovulation was triggered with human chorionic gonadotropin (hCG) or GnRH agonists when appropriate.

Transvaginal ultrasound and serum estradiol (E2) and progesterone (PRG) assessments were used to monitor follicular development and to exclude premature luteinization. Oocyte retrieval was performed 34–38 h after ovulation triggering.

Retrieved oocytes were assessed for maturity by experienced embryologists. Metaphase II (MII) oocytes were selected and cryopreserved using vitrification method according to standard laboratory protocols.

### Data collection and outcomes

Baseline characteristics were collected including key data related to first COS cycle and, for DuoStim patients, corresponding LPS data. These included age, serum AMH level (Elecsys® assay, Roche), AFC, serum E2 and PRG levels on trigger day, and COS duration and medication information. In particular, AMH testing was routinely performed at the first FP consultation, with a mean ± SD interval of 47 ± 47 days and a median of 45 days (IQR: 6–74) before treatment.

### Primary outcome

Was comparison between cohorts in number of retrieved (RO) and mature MII vitrified (VO) oocytes in patients undergoing conventional COS.

### Secondary outcome

Was the same comparison in the DuoStim subgroup.

### Statistical analysis

Descriptive statistics were calculated for patient and treatment characteristics. Data were summarized using means and standard deviations or medians with interquartile ranges according to distribution. Normality was assessed using Shapiro–Wilk test. Comparisons between AMH cohorts used Student’s *t*-test for normally distributed variables and Mann–Whitney *U* test for non-normally distributed variables. Categorical variables were compared using chi-square test**.**

Ovarian stimulation efficiency was evaluated using RO per 1000 IU of total gonadotropin dose and VO per 1000 IU of total gonadotropin dose. Vitrification process efficiency was assessed by calculating VO to RO ratio (VO/RO ratio).

Correlations between continuous variables were evaluated using Spearman’s correlation coefficient.

ANCOVA was conducted with VO as the dependent variable, AMH cohort as a  fixed factor, and age as a covariate.

Multiple linear regression analyzed combined effect of AMH levels and age on VO.

All analyses were performed using IBM SPSS Statistics Version 29.0 (IBM Corp., Armonk, NY, USA). A two-tailed *p*-value < 0.05 was considered statistically significant.

## Results

A total of 801 women attended the initial FP consultation. Of these, 407 were excluded for not proceeding with FP, 88 for missing AMH measurement, and 2 for age over 40. A total of 304 patients were included (49 in cohort 1, 255 in cohort 2).

Baseline characteristics are described in Table 1. The indication for FP in cohort 1 was DOR, defined by AMH ≤ 0.5 ng/mL, without identifiable etiological factors. Cohort 2 indications included cancer (*n* = 140, 54.9%), endometriosis (*n* = 43, 16.9%), benign conditions requiring gonadotoxic treatments (*n* = 6, 2.3%), POI-associated genetic conditions and BRCA mutation without cancer history (*n* = 5, 2.0%), previous ovarian surgery or gonadotoxic treatment (*n* = 23, 9.0%), and elective oocyte cryopreservation (*n* = 38, 14.9%).

**Table 1:** Baseline characteristics of the included population, stratified by cohort

	Cohort 1 (DOR)				Cohort 2 (control group)				
	*N*	Mean ± SD	Median (IQR)	Range	*N*	Mean ± SD	Median (IQR)	Range	*p*
**Age**	49	32.4 ± 5.6	33.0 (29.5–37.0)	19.0–40.0	255	31.4 ± 5.5	32.0 (28.0–36.0)	16.0–40.0	0.214
AMH***	49	0.30 ± 0.13	0.32 (0.19–0.41)	0.04–0.5	255	2.55 ± 2.18	1.96 (1.01–3.41)	0.51–13.78	<.001
AFC***	49	4.6 ± 1.9	4.0 (3.0–6.0)	1.0–9.0	255	12.7 ± 7.6	11.0 (7.0–16.0)	1.0–40.0	< 0.001

AMH (ng/mL); Age (years); AFC (*n*); ^***^p < 0.001

Cohort 1: women with severely diminished ovarian reserve (serum AMH ≤ 0.5 ng/mL); Cohort 2: women with normal to reduced ovarian reserve (serum AMH > 0.5 ng/mL)

No significant differences were observed between cohorts in age (32.4 ± 5.6 vs. 31.4 ± 5.5 years, *p* = 0.214), and stimulation duration (9.9 ± 2.3 vs. 10.0 ± 1.9, *p* = 0.657).

The two cohorts differed in several measures (Tables 1 and 2): AFC (4.6 ± 1.9 vs. 12.7 ± 7.6, *p* < 0.001), daily gonadotropin dosages (394.5 ± 62.8 IU/day, range 150–450 IU/day, vs. 275.33 ± 92.2 IU/day, range 75–450 IU/day, *p* < 0.001), total gonadotropin dosages (3841.0 ± 1175.7 IU, range 900–6300 IU, vs. 2746.1 ± 1100.2 IU, range 975–7200 IU, *p* < 0.001), and E2 levels at trigger (1003.3 ± 576.7 vs. 1841.8 ± 1364.2 pg/mL, *p* < 0.001).

**Table 2:** Stimulation characteristics and cycle outcomes, stratified by cohort

	Cohort 1 (DOR)						Cohort 2 (control group)		
	*N*	Mean ± SD	Median (IQR)	Range	*N*	Mean ± SD	Median (IQR)	Range	*p*
**Stim Dur**	49	9.9 ± 2.3	10.0 (8.0–12.0)	6.0–15.0	255	10.0 ± 1.9	10.0 (9.0–11.0)	4.0–18.0	0.657
Daily Gn***	49	394.5 ± 62.8	375.0 (375.0–450.0)	150.0*–*450.0	255	275.3 ± 92.2	255.8 (208.8–347.0)	75.0–450.0	< 0.001
Tot Gn***	49	3841.0 ± 1175.7	3750.0 (2775.0–4800.0)	900.0–6300.0	255	2746.1 ± 1100.2	2550.0 (1925.0–3375.0)	975.0–7200.0	< 0.001
E2***	49	1003.3 ± 576.7	840.0 (638.4–1172.5)	118.0–3230.0	255	1841.8 ± 1364.2	1404.5 (945.3–2294.0)	161.0–8076.0	< 0.001
**PRG**	49	1.0 ± 0.8	0.6 (0.5–1.2)	0.1–4.2	255	1.3 ± 1.6	0.9 (0.5–1.3)	0.1–13.6	0.134
RO***	49	3.1 ± 2.3	3.0 (1.0–4.0)	0.0–12.0	255	9.0 ± 6.5	8.0 (5.0–12.0)	0.0–40.0	< 0.001
VO***	49	2.3 ± 1.9	2.0 (1.0–3.0)	0.0–9.0	255	7.4 ± 5.1	6.0 (4.0–10.0)	0.0–28.0	< 0.001
**VO/RO**	46	75.1 ± 35.9	100.0 (66.7–100.0)	0.0–100.0	248	84.1 ± 17.5	87.5 (74.0–100.0)	0.0–100.0	0.901

*Stim Dur*, stimulation duration (days); *daily G*n, daily gonadotropin dose (IU); *total Gn*, total gonadotropin dose (IU); E2 (pg/mL); PRG (ng/mL); *RO*, retrieved oocytes (*n*); *VO*, vitrified oocytes (*n*); *VO/RO*, VO/RO ratio (%); ^***^p < 0.001

Cohort 1 includes women with severely diminished ovarian reserve, defined as serum AMH levels ≤ 0.5 ng/mL

Cohort 2 includes women with normal to reduced ovarian reserve, defined as serum AMH levels > 0.5 ng/mL

Oocyte yield was significantly different between cohorts for both RO (3.1 ± 2.3 vs. 9.0 ± 6.5, *p* < 0.001) and VO (2.3 ± 1.9 vs. 7.4 ± 5.1, *p* < 0.001).

Cohort 1 showed significantly lower stimulation efficiency compared to cohort 2 for both RO per 1000 IU of total gonadotropin (mean rank 49.0 vs. 126.3; *p* < 0.001) and VO per 1000 IU of total gonadotropin (mean rank 49.3 vs. 126.2; *p* < 0.001).

**Fig. 2:**
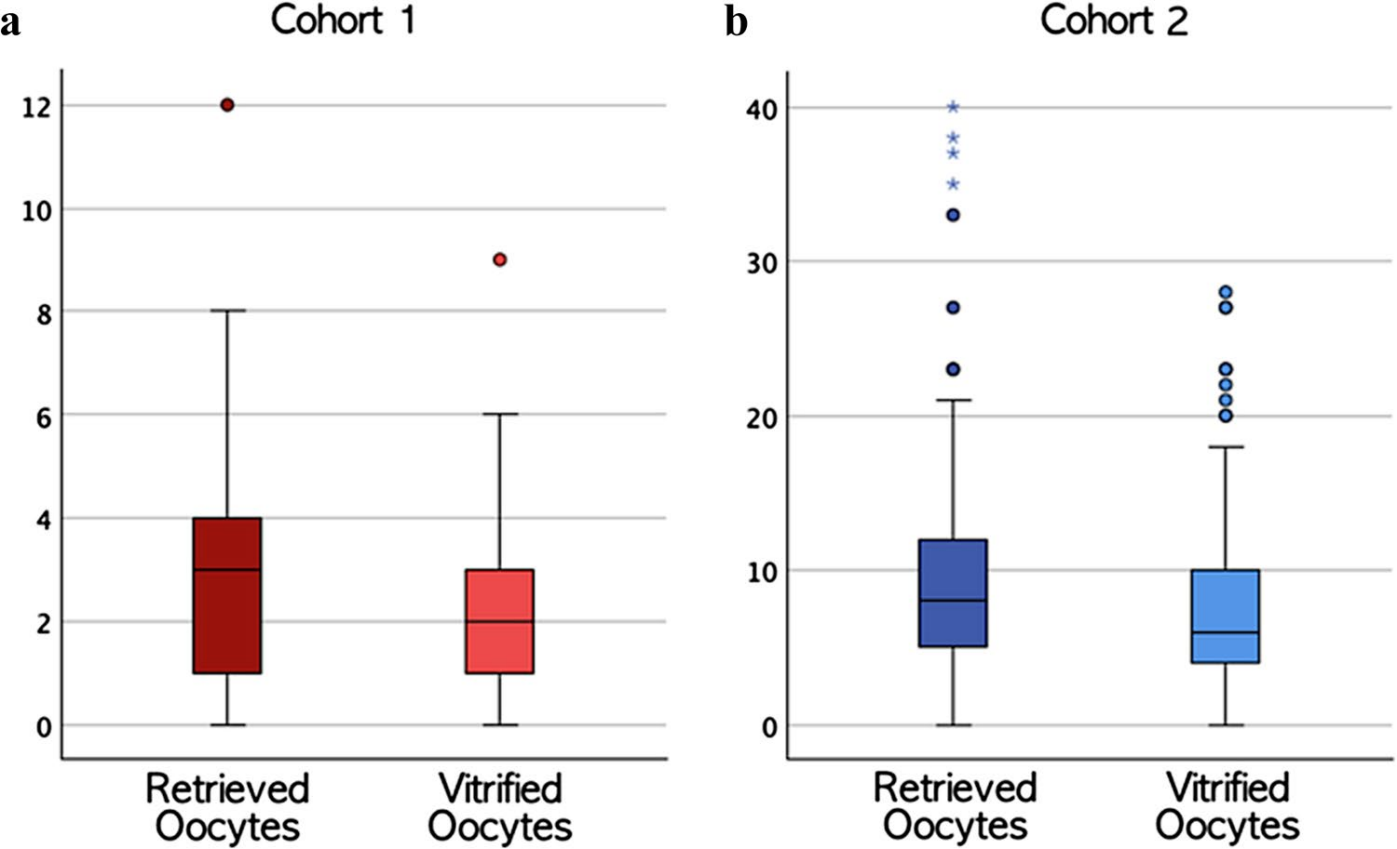
**a**, **b** Oocyte outcomes by AMH cohort. Box plots comparing retrieved and vitrified oocyte yields between (A) cohort 1 (AMH ≤ 0.5 ng/mL, red) and (B) cohort 2 (AMH > 0.5 ng/mL, blue). Cohort 1 showed significantly lower outcomes for both retrieved oocytes (mean ± SD: 3.1 ± 2.3, median 3.0, IQR 1.0–4.0) and vitrified oocytes (mean ± SD: 2.3 ± 1.9, median 2.0, IQR 1.0–3.0) compared to cohort 2 (retrieved: mean ± SD: 9.0 ± 6.5, median 8.0, IQR 5.0–12.0; vitrified: mean ± SD: 7.4 ± 5.1, median 6.0, IQR 4.0–10.0, both *p* < 0.001). Box plots display median, interquartile ranges, whiskers representing 1.5 × IQR, and outliers as individual points

No significant difference was observed in oocyte vitrification process efficiency between cohorts. In cohort 1, mean VO/RO ratio was 75.1% ± 35.9% (median 100.0%, IQR 66.7–100.0%). In cohort 2, mean VO/RO ratio was 84.1% ± 17.5% (median 87.5%, IQR 74.0–100.0) (Fig. 2 a,b).

Strong positive correlations were found between AMH levels and RO (*ρ* = 0.636, *p* < 0.001) and VO (*ρ* = 0.624, *p* < 0.001). AMH showed a negative correlation with age (*ρ* =  − 0.110, *p* = 0.05). Age showed negative correlations with RO (*ρ* =  − 0.179, *p* = 0.002) and VO (*ρ* =  − 0.199, *p* < 0.001).

ANCOVA showed significant independent effect of AMH cohort on VO after age adjustment (*F*(1, 301) = 44.97, *p* < 0.001, partial *η*^2^ = 0.130). Age had a significant independent effect on VO yield (*F*(1,301) = 10.19, *p* = 0.002, partial *η*^2^ = 0.033). The model explained 16.2% of the variance (*F*(2,301) = 29.09, *p* < 0.001).

Linear regression confirmed as significant predictors of VO both AMH (*B* = 1.151, SE = 0.117, *β* = 0.487, *p* < 0.001) and age (*B* =  − 0.139, SE = 0.046, *β* =  − 0.151, *p* = 0.002) (*F*(2, 301) = 56.31, *p* < 0.001, explaining 27.2% of the variance.

### Secondary outcome

Thirty-eight patients underwent DuoStim (12 in cohort 1, 26 in cohort 2). Baseline characteristics are described in Tables 3 and 4.

**Table 3:** Baseline characteristics of the DuoStim subgroup

Cohort 1 (DOR)						Cohort 2 (control group)			
	*N*	Mean ± SD	Median (IQR)	Range	*N*	Mean ± SD	Median (IQR)	Range	*p*
**Age**	12	30.3 ± 6.0	32.0 (26.3–34.0)	20.0–40.0	26	32.2 ± 5.3	32.5 (29.8–35.3)	19.0–40.0	0.395
AMH***	12	0.22 ± 0.10	0.19 (0.14–0.30)	0.1–0.44	26	1.36 ± 1.23	0.94 (0.64–1.55)	0.51–5.12	< 0.001

Age (years); AMH (ng/mL); ^***^p < 0.001

Cohort 1 includes women with severely diminished ovarian reserve, defined as serum AMH levels ≤ 0.5 ng/mL

Cohort 2 includes women with normal to reduced ovarian reserve, defined as serum AMH levels > 0.5 ng/mL

**Table 4:** DuoStim outcomes, stratified by cohort

	Cohort 1 (DOR)				Cohort 2 (control group)				
	*N*	Mean ± SD	Median (IQR)	Range	*N*	Mean ± SD	Median (IQR)	Range	*p*
**FPS RO**	12	2.5 ± 1.8	2.5 (1.0–4.0)	0.0–6.0	26	4.5 ± 2.6	4.0 (3.0–6.0)	0.0–11.0	0.023
Tot RO**	12	4.1 ± 2.4	4.0 (1.5–6.0)	1.0–8.0	26	8.1 ± 4.1	7.5 (6.0–10.3)	2.0–22.0	.001
**FPS VO**	12	1.9 ± 1.6	1.5 (1.0–3.0)	0.0–5.0	26	3.8 ± 2.8	3.0 (1.0–6.0)	0.0–10.0	0.047
Tot VO**	12	3.3 ± 2.1	3.5 (1.0–5.5)	0.0–6.0	26	6.9 ± 3.3	7.0 (4.8–9.0)	2.0–15.0	.001

Age (years); AMH (ng/mL); *FPS RO*, retrieved oocytes from follicular phase stimulation (*n*); *Tot RO*, total retrieved oocytes from follicular and luteal phase stimulation (*n*); *FPS VO*, vitrified oocytes from follicular phase stimulation (*n*); *Tot VO*, total vitrified oocytes from follicular and luteal phase stimulation (*n*); ^**^p < 0.01

Cohort 1 includes women with severely diminished ovarian reserve, defined as serum AMH levels ≤ 0.5 ng/mL

Cohort 2 includes women with normal to reduced ovarian reserve, defined as serum AMH levels > 0.5 ng/mL

FPS oocyte yields were significantly lower in cohort 1 compared to cohort 2: FPS RO in cohort 1 2.5 ± 1 vs. in cohort 2 4.5 ± 2.6 (*p* = 0.023); FPS VO in cohort 1 1.9 ± 1.6 vs. in cohort 2 3.8 ± 2.8 (*p* = 0.047).

Following FPS and LPS, total RO and total VO remained significantly lower in cohort 1 compared to cohort 2: total RO in cohort 1 4.1 ± 2.4 vs. in cohort 2 8.1 ± 4.1 (*p* = 0.001); total VO in cohort 1 3.3 ± 2.1 vs. in cohort 2 6.9 ± 3.3 (*p* = 0.001).

## Discussion

To our knowledge, this is the first study specifically addressing FP in women with severely reduced ovarian reserve, an extremely challenging and under-investigated population. While previous studies about POR have focused on stimulation strategies and oocyte yield in ART [16, 19, 20, 23], few have investigated outcomes in low-AMH women within a FP setting.

International guidelines recommend timely FP counseling for women at risk of POI, recognizing that in many cases follicular depletion may already preclude effective intervention [22]. FP may nonetheless be useful in preserving reproductive autonomy and reducing reliance on donor gametes, with important ethical and economic implications.

Limited data exist on optimal numbers of cryopreserved mature oocytes needed for live birth. To achieve cumulative live birth rates (LBR) of 40–70%, 10–15 oocytes may be required in women ≤ 35 years of age, with higher numbers required as age increases [6, 8]. However, acceptable rates (30–45%) can occur with 8–10 oocytes [5]. For women with DOR, these targets are rarely achievable.

Our findings provide novel, real-world evidence to guide FP eligibility and counseling and to support the development of dedicated stratification frameworks for FP—analogous to those used in ART but adapted to its specific clinical objectives—moving from a binary eligibility model to a graded, personalized counseling framework.

Our analysis reinforces AMH as a robust predictor of ovarian response. In our study, women with AMH ≤ 0.5 ng/mL achieved oocyte yields consistently below clinically meaningful thresholds, in contrast to women with higher AMH levels, who exhibited significantly better responses to stimulation. This discrepancy persisted even with DuoStim protocols: patients with AMH > 0.5 ng/mL achieved clinically meaningful cumulative yield, gaining on average three additional oocytes, whereas those with AMH ≤ 0.5 ng/mL gained only one. This yield falls substantially below the number of mature oocytes suggested for reasonable LBR [5, 6, 8, 18].

Our results also confirm that increasing gonadotropin doses offers minimal benefit in DOR, consistent with evidence that doses above 300 IU/day provide limited additional improvement [2, 21]. This reflects biological limitations rather than suboptimal stimulation protocols. Laboratory efficiency was high and comparable between cohorts, excluding laboratory performance as a confounder.

Age exhibited the expected negative correlation with VO and AMH. The significant independent age effect observed in our analysis, consistent with prior evidence [8, 11, 13, 18, 25], supports age-stratified approaches. Therefore, the selective application of age-related approaches for DOR may represent a viable strategy for achieving clinically meaningful reproductive opportunities that justify FP efforts.

### Limitations

This study has several limitations inherent to its retrospective design, including potential selection and information bias. The DuoStim subgroup was small and not treated electively; despite satisfactory post hoc statistical power for key outcomes, its limited size and exploratory nature restrict generalizability. Temporal variations in clinical practice and the small number of DOR patients limited subgroup analyses and prevented evaluation of age-related differences. The primary endpoint was oocyte yield, but no follow-up data were available on oocyte warming, fertilization, or live birth. Larger, prospective studies are needed to validate these findings and refine predictive criteria for FP outcomes in DOR populations.

### Clinical implications and future directions

Clinicians should provide realistic, evidence-based counseling, emphasizing that protocol optimization cannot overcome the intrinsic limitations of an exhausted follicular pool.

Non-specialist physicians should be encouraged to assess ovarian reserve, at least through AMH testing, and to recognize warning signs of DOR, such as family history of POI, menstrual irregularities, or POI-associated conditions, ensuring timely FP referral.

The implications of our study extend beyond individual care to strategic resource allocation for FP programs, particularly those employing AMH-based eligibility criteria.

Further research should clarify the role of DuoStim and age-stratified approaches, and develop multi-parameter prediction models to improve prognostic categorization and individualized counseling. The complex interplay of genetic, hormonal, lifestyle, and individual physiological factors demands a multidimensional approach to personalize FP strategies.

Integration of cost-effectiveness assessments analyses and long-term outcome registries will be essential to guide sustainable, ethic and evidence-based FP implementation.

Moreover, future studies are needed to investigate FP outcomes in women with DOR and identifiable etiological factors, as these subgroups may differ in both response to stimulation and prognosis.

## Conclusion

Women with AMH ≤ 0.5 ng/mL achieve inadequate oocyte yields for successful FP, even with intensive protocols like DuoStim, substantially compromising live birth potential and questioning the clinical utility of FP in this population.

Age may represent an important factor within this compromised population: younger women with severe DOR might still have a chance to benefit, potentially representing the subgroup with the most realistic FP potential.

These findings may contribute to developing evidence-based patient selection systems for FP. The broader implications extend to FP programs design and healthcare policy, requiring careful reconsideration of AMH-based eligibility criteria and resource allocation strategies.

Early identification of at-risk patients is essential. Our findings suggest that revising AMH thresholds upward and enhancing clinicians’ awareness may facilitate timely referral for definitive counseling at an appropriate time.

This work points towards a new era of FP personalization, laying the groundwork for future research aimed at distinguishing low-AMH subpopulations most likely to benefit from FP. Prospective studies with larger cohorts are needed to validate precise age cut-offs and additional predictive factors to identify DOR patients with realistic chances of FP success.

The FP community should embrace an evidence-based approach that balances compassionate care with clinical pragmatism, acknowledging biological realities while ensuring patients receive care founded on realistic expectations rather than misplaced optimism.

## Data Availability

Data regarding any of the subjects in the study has not been previously published unless specified. The datasets used and/or analyzed during the current study are available from the corresponding author on reasonable request.
